# Small incision lenticule extraction (SMILE) versus laser in-situ keratomileusis (LASIK): study protocol for a randomized, non-inferiority trial

**DOI:** 10.1186/1745-6215-13-75

**Published:** 2012-05-31

**Authors:** Marcus Ang, Donald Tan, Jodhbir S Mehta

**Affiliations:** 1 Singapore National Eye Centre, Singapore, Singapore; 2 Singapore Eye Research Institute, Singapore, Singapore; 3 Department of Ophthalmology, National University Health System, Singapore, Singapore; 4 Department of Clinical Sciences, Duke-NUS Graduate Medical School, Singapore, Singapore

**Keywords:** Refractive surgery, Laser in situ keratomileusis, Small incision lenticule extraction

## Abstract

**Background:**

Small incision lenticule extraction or SMILE is a novel form of ‘flapless’ corneal refractive surgery that was adapted from refractive lenticule extraction (ReLEx). SMILE uses only one femtosecond laser to complete the refractive surgery, potentially reducing surgical time, side effects, and cost. If successful, SMILE could potentially replace the current, widely practiced laser in-situ keratomileusis or LASIK. The aim of this study is to evaluate whether SMILE is non-inferior to LASIK in terms of refractive outcomes at 3 months post-operatively.

**Methods/Design:**

Single tertiary center, parallel group, single-masked, paired-eye design, non-inferiority, randomized controlled trial. Participants who are eligible for LASIK will be enrolled for study after informed consent. Each participant will be randomized to receive SMILE and LASIK in each eye. Our primary hypothesis (stated as null) in this non-inferiority trial would be that SMILE differs from LASIK in adults (>21 years old) with myopia (> −3.00 diopter (D)) at a tertiary eye center in terms of refractive predictability at 3 months post-operatively. Our secondary hypothesis (stated as null) in this non-inferiority trial would be that SMILE differs from LASIK in adults (>21 years old) with myopia (> −3.00 D) at a tertiary eye center in terms of other refractive outcomes (efficacy, safety, higher-order aberrations) at 3 months post-operatively. Our primary outcome is refractive predictability, which is one of several standard refractive outcomes, defined as the proportion of eyes achieving a postoperative spherical equivalent (SE) within ±0.50 D of the intended target. Randomization will be performed using random allocation sequence generated by a computer with no blocks or restrictions, and implemented by concealing the number-coded surgery within sealed envelopes until just before the procedure. In this single-masked trial, subjects and their caregivers will be masked to the assigned treatment in each eye.

**Discussion:**

This novel trial will provide information on whether SMILE has comparable, if not superior, refractive outcomes compared to the established LASIK for myopia, thus providing evidence for translation into clinical practice.

**Trial registration:**

Clinicaltrials.gov NCT01216475.

## Background

Laser in-situ keratomileusis (LASIK) is the current laser refractive procedure of choice to treat myopia. The advantages of LASIK include early postoperative improvement and stabilization of visual acuity, minimal postoperative patient discomfort, and the possibility of enhancement in the future [1]. However, side effects such as dry eyes, reduced vision in low lighting conditions, and visual distortions such as glare and haloes can still occur in up to 1% to 2% of cases; while flap-related complications, inflammation, or infection, though rare, can have serious consequences [2,3].

Femtosecond lasers have been widely used in LASIK to fashion a corneal flap, which is followed by corneal ablation using a separate excimer laser [4]. The advantages of femotosecond or ‘bladeless’ LASIK over microkeratome LASIK flaps are reduced postoperative dry-eye symptoms, reduced likelihood of flap dislocation, and reduced incidence of buttonholes or free caps [2,3]. Recently, refractive lenticule extraction (ReLEx) has been introduced as a single laser refractive procedure without the use of an excimer laser [5-8]. Small incision lenticule extraction (SMILE) is a variation of ReLEx that requires no retractable flap, thus reducing surgical time, reduced patient inconvenience from moving from one laser to another, and, potentially, more accurate ablation [8].

A review of current literature reveals that there are few studies available to validate the outcome of ReLEx or SMILE, which has obtained CE Mark approval in 2009 but yet to be approved by the United States FDA [5-8]. Initial clinical results have been promising, which found that postoperative refractive outcomes after ReLEx were comparable to LASIK with few complications [9,10]. However, there are currently no randomized controlled trials comparing both surgical procedures. We have also conducted preliminary clinical and experimental studies with promising results and confirmed the safety and efficacy of ReLEx [11].

Non-inferiority trials are used to compare standard treatment with a new treatment that is expected to have some advantages such as greater predictability, less side effects, or greater improvement in quality of life [12,13]. LASIK, which is the current gold standard for corneal refractive surgery, already produces good visual outcomes with refractive predictability. As we do not expect to see a great improvement to the results from the already established LASIK, we aim to demonstrate that SMILE is just as good in terms of visual outcome in this randomized non-inferiority trial.

## Methods/Design

### Study objective and hypotheses

In this study we aim to demonstrate that the refractive predictability of SMILE is not inferior to the established procedure (LASIK) to treat myopia. Refractive predictability is one of several standard refractive outcomes, defined as the proportion (number) of eyes achieving a postoperative spherical equivalent (SE) within ±0.50 diopter (D) of the intended target. Our primary hypothesis (stated as null) in this non-inferiority trial would be that SMILE differs from LASIK in adults (>21 years old) with myopia (> −3.00 D) at a tertiary eye center in terms of refractive predictability at 3 months postoperatively. Our secondary hypothesis (stated as null) in this non-inferiority trial would be that SMILE differs from LASIK in adults (>21 years old) with myopia (> −3.00 D) at a tertiary eye center in terms of other refractive outcomes (efficacy, safety, higher-order aberrations) at 3 months postoperatively.

### Trial design

This trial is a single tertiary center, parallel group, single-masked, paired-eye design, non-inferiority, randomized controlled trial. We will use a paired-eye study design, with subjects randomly assigned to undergo one procedure in each eye (SMILE in one eye, LASIK in the other eye). All procedures will be performed in the SNEC Refractive Center by the same fully qualified refractive surgeons, who are co-investigators in this study. Each surgeon has performed more than 30 similar surgeries and 20 cases of SMILE to ensure that each surgeon is adept at performing this procedure. After randomization and random allocation to treatment group, each subject will undergo either SMILE or LASIK in one eye, followed by LASIK or SMILE in the fellow eye on the same day (either the left or right eye will be randomized to decide which eye is operated on first). We have obtained ethics approval from our Institutional Review Board (CIRB Ref No: 2011/109/A) and this trial has been registered (Clinical Trials Registration NCT01216475).

### Participants and recruitment

All participants with bilateral myopia will be recruited at the Singapore National Eye Center (SNEC) with the inclusion and exclusion criteria detailed in Table 1. All subjects will be recruited and provide written informed consent that explains the details of the trial, interventions, and study protocol in accordance with the principles of the Declaration of Helsinki.

**Table 1:** Inclusion and exclusion criteria for trial participants

**Inclusion criteria**	**Exclusion criteria**
I. 21 years of age or older	I. Progressive or unstable myopia and/or astigmatism
II. Cycloplegic spherical equivalent of >−3.00 D in BOTH eyes	II. Clinical or corneal topographic evidence of keratoconus
III. Refractive cylinder −2.00 D or less in BOTH eyes and anisometropia of <1D comparing BOTH eyes	III. Residual, recurrent or active ocular disease such as uveitis, severe dry eyes, severe allergic eye disease, glaucoma, visually significant cataract, and retinal disease
IV. Best spectacle corrected visual acuity (BSCVA) of 6/12 or better in BOTH eyes	IV. Previous corneal surgery or trauma within the corneal flap zone
V. Spherical or cylindrical error has progressed at −0.50 D or less per year from date of baseline measurement in BOTH eyes	V. Patent corneal vascularization within 1 mm of the corneal flap zone
VI. Contact lens wearers must have removed contact lenses at least 2 weeks before the baseline measurement	VI. Taking systemic medications likely to affect wound healing, such as corticosteroids and antimetabolites. Systemically immunocompromised or systemic disease likely to affect wound healing, such as diabetes, connective tissue disease, and severe atopy; pregnant or nursing
VII. No evidence of irregular astigmatism on corneal topography in BOTH eyes	

### Surgical interventions

#### SMILE procedure

Each SMILE procedure will be performed using an established, described technique [7]. After application of topical anesthesia, standard sterile draping, and insertion of the speculum, the patient’s eye will be centered and docked with the curved interface cone before application of suction fixation. The laser will then be activated for photo-dissection in the following sequence: first the posterior surface of the refractive lenticule (spiral in), then the lenticule border is created. The anterior surface of the refractive lenticule (spiral out) is then formed which extended beyond the posterior lenticule diameter by 0.5 mm to form the anterior flap and is followed by a rim cut. We will use the following FS laser parameters: 120 μm flap thickness, 7.5 mm flap diameter, 6.5 mm optical zone of lenticule, 145 nj of power with side cut angles at 90°. A superior hinge, 50° in cordal length, will be made in all cases. The spot distance and tracking spacing are 3/3 μm for the lenticule, 2.5/2.5 μm for the lenticule side cut, 3/3 μm for the flap, and 2/2 μm for the flap side cut. After the suction is released, a Siebel spatula (Rhein Medical, Heidelberg, Germany) is inserted under the flap near the hinge before the flap is separated and reflected. The edge of the refractive lenticule is separated from the stromal bed with a sinsky hook and the posterior border of the lenticule gently separated with the Siebel spatula. The lenticule is then grasped with non-toothed serrated forceps through the small incision.

#### LASIK procedure

Each LASIK procedure will be performed using a standard, established technique. Under topical anesthesia and standard draping, a lid speculum is used to retract the eyelids, and polyvinyl acetate surgical spears (Ivalon, New London, CT) to dry the conjunctival fornices. A superiorly hinged 120/140 μm thick flap will be created using the Visumax femtosecond laser (Carl Zeiss). Excimer laser ablation is then performed using Wavelight Allegretto WAVE Eye-Q 400 Hz excimer laser (Wavelight GmbH, Alcon, Fort Worth, TX, USA). After ablation, the flap will be carefully repositioned, and postoperative medications are commenced.

### Outcomes

We plan to use standard primary and secondary outcomes measures at 3 months postoperatively, which are reported in any assessment refractive surgical technique and standard outcomes in refractive studies. Measurements and outcomes are based on visual acuity (VA) and refraction that are performed by trained refractive optometrists and are repeatedly tested to ensure accuracy and reproducibility. Our primary outcome measure is refractive predictability, which is defined as the proportion number of eyes achieving a postoperative spherical equivalent (SE) within ±0.50 D of the intended target.

Secondary outcome measures include: (1) Efficacy: defined as the proportion number of eyes achieving a unaided visual acuity (UAVA) of 20/20 or better postoperatively; (2) Safety: defined as the proportion number of eyes that lost or gained one or more lines of postoperative best-corrected visual acuity (BCVA) relative to the preoperative BCVA; (3) Higher-order aberrations (HOAs): measured using the Bausch and Lomb Technolas Zywave aberrometer with Zywave software version 4.45 (ZYOPTIX Diagnostic Workstation, Bausch & Lomb); (4) Contrast sensitivity: tested using the Vision Contrast Test System (VCTS) chart (VCTS 6500 contrast sensitivity chart) in six spatial frequencies.

### Sample size

As this is a paired design, non-inferiority trial with a binary outcome, we calculate the required sample size using the maximum likelihood method for large sample proposed by Nam (1997) [14]. A review of current literature reveal that the reported refractive predictabilities in LASIK and SMILE range from 78.2% to 96.7% [1,14,15] and from 90.0% to 95.6%, [5-8], respectively. The results from our own audit department estimate our refractive predictability at 82% for LASIK (2011, unpublished). We therefore assumed the refractive predictabilities in LASIK and SMILE in this study are 82% and 92%, respectively. Thus, a sample of 67 subjects (134 eyes) will be sufficient to confirm non-inferiority with a power of ≥80% and at a 5% significance level using a 10% non-inferiority margin, which is the clinically significant difference from our preliminary data. To account for a lost to follow-up rate of 5%, 70 subjects will be recruited.

### Randomization and blinding

The random allocation sequence will be generated by a computer with no blocks or restrictions, and implemented by concealing the number-coded surgery within sealed envelopes until just before the procedure. This randomization process will be performed by a research assistant masked to the study subjects and participants will be enrolled by co-investigator surgeons who will assign participants to their groups after opening the sealed envelope, that is each subject will receive a different procedure in each eye at random. In this single-masked trial, subjects and their caregivers will be masked to the assigned treatment in each eye. Both procedures will be performed within the SNEC Refractive Suite, using the femtosecond laser machine. Clinically, it is impossible to detect any difference between each procedure postoperatively to the untrained eye without the slit-lamp microscope. While the surgeons cannot be masked as they will be performing the intervention, the outcome assessors such as nurses, research assistants, and trained optometrists will also be masked to the assigned treatment to improve the objectivity of the research outcomes, as well as to minimize bias. In the event of adverse events (please see below), a code-breaking envelope for each subject will be available.

### Data collection

All patients will have data collection forms outlining each follow-up visit and data to be collected at each visit, which include visual acuity, refraction results, clinical examination findings, and the outcome measures as described above. All data will be securely stored in the SNEC Refractive Suite and then entered into a password-secure desktop computer locked within the Singapore Eye Research Institute, with data back-up into hard drives done daily. Only the named investigators will have access to the research data. All data access will be monitored and controlled by the PI. At the end of the study, the research data will be entered by the research assistant and stored for up to 5 years in compliance with any integrity issues that may arise from any subsequent publications. Following that time period the data will be kept under the control of the PI.

### Adverse events

All subjects will be monitored during enrolment into the study for adverse events. All adverse events or serious adverse event (SAE) will be reported to both the centralized institution review board and institution heads (Singhealth) according to the guidelines (http://research.singhealth.com.sg).

### Statistical analyses

Demographic and baseline information will be described, and eye-specific characteristics will be described for each treatment. To study the non-inferiority of SMILE to LASIK, a 90% confidence interval of the difference in predictability between the two treatments (LASIK minus SMILE) will be constructed by a method using score intervals with continuity correction (method #10 in Newcombe RG, 1998) [15]. If the upper limit of the 90% confidence interval does not exceed the pre-defined non-inferiority margin of 5%, non-inferiority is confirmed. Similarly, for each of the two secondary outcomes, efficacy, and safety, a 90% confidence interval of the difference between the two treatments using the above-mentioned method will be constructed and then compared with a non-inferiority margin of 5%. Assuming the other secondary outcome, HOA, follows a normal distribution, a 90% confidence interval of the difference between the two treatments will be constructed through a paired *t*-test, and then compared with a non-inferiority margin of 10%. We would not be performing any interim analyses due to the short duration of follow-up for each outcome measure.

## Discussion

In this non-inferiority trial, we aim to demonstrate that SMILE is just as good as LASIK in terms of refractive outcome, as we do not expect to see a great improvement to the results from the already established LASIK procedure. Moreover, this trial may show that SMILE has additional benefits, such as reduction in higher-order aberrations that leads to better quality of vision. On the other hand if we use a superiority trial design with a small sample size that fails to demonstrate any difference between LASIK and SMILE, would be inconclusive since it does not necessarily prove equivalence. Thus we use a non-inferiority trial design to compare our primary and secondary outcomes.

Despite its proven efficacy, LASIK still requires the use of two laser machines: one for the flap creation and another for the excimer ablation. This increases the cost as well as the surgical time for the procedure. SMILE uses only one laser machine, thus potentially reduces surgical time and cost. Moreover, SMILE does not involve any flap creation, which potentially reduces risk of side effects such as dry eyes. For these reasons, SMILE is potentially a new, improved form of refractive surgery, which may supersede LASIK and change clinical practice. There is also potentially a positive impact on healthcare as surgical time and costs are reduced with this new, ‘all-in-one’ laser procedure. Moreover, the needs for enhancements or retreatment are higher in patients with high myopia, a common condition in Singapore. SMILE can be used to treat high myopia without the extra costs involved in visual rehabilitation following LASIK enhancement.

More importantly, in the longer term we propose that SMILE may potentially develop into a reversible surgical procedure. Unlike LASIK which uses an excimer laser to ablate or destroy corneal tissue, SMILE cuts and removes a piece of corneal lenticule, which may be stored and replaced into the cornea at a later time. This is important as we can potentially reverse the refractive procedure many years later when the patient’s myopia decreases and presbyopia sets in. The ability to re-implant the cornea lenticule allows for treatment of corneal ectasia, reversal or monovision, or even the possibility of a presbyopic implant. We have done preliminary studies to demonstrate corneal lenticule viability after ReLEx [16]; and in an experimental animal study, we demonstrated proof of principle of a reversible corneal refractive procedure for the first time (unpublished). We were able to successfully store and re-implant an autologous stromal lenticule into rabbit eyes with minimal resultant inflammation and no signs of rejection after 28 days. This is important as we can potentially reverse the refractive procedure when the patient develops presbyopia. The ability to re-implant the cornea lenticule allows for treatment of corneal ectasia, reversal of myopia, monovision or even the possibility of a presbyopic implant.

In conclusion, this non-inferiority clinical trial that compares SMILE and LASIK will help to determine if this new refractive procedure, SMILE, has equal or better visual and refractive outcomes compare to the traditional LASIK for treatment of myopia. Results of this trial will likely impact clinical practice with potentially further development into novel techniques for re-implantation and reversibility.

### Trial status

Ongoing.

## Abbreviations

LASIK, Laser in-situ keratomileusis; ReLEx, Refractive lenticule extraction; SMILE, Small incision lenticule extraction.

## Competing interests

The authors declare that they have no competing interests.

## Authors’ contributions

All authors (MA, DT, JSM) participated in the design of the study, performed the statistical analysis, participated in its coordination, and helped to draft the manuscript. All authors read and approved the final manuscript.
